# Interdisciplinary in situ simulation-based medical education in the emergency department of a teaching hospital in Nepal

**DOI:** 10.1186/s12245-019-0235-x

**Published:** 2019-08-27

**Authors:** Roshana Shrestha, Anmol Purna Shrestha, Sanu Krishna Shrestha, Samjhana Basnet, Alok Pradhan

**Affiliations:** Department of General Practice and Emergency Medicine, Kathmandu University School of Medical Sciences, Dhulikhel, Kavrepalanchok Nepal

**Keywords:** Defibrillator, In situ simulation, Latent safety threats, Simulation-based medical education, Teamwork

## Abstract

**Background:**

Simulation is well established as an effective strategy to train health care professionals in both technical and nontechnical skills and to prevent errors. Despite its known efficacy, adequate implementation is restricted due to the financial burden in resource-limited settings like ours. We therefore pursued to introduce cost-effective in situ simulation (ISS) in the emergency department (ED) to explore its impact on perception and learning experience among multidisciplinary health care professionals and to identify and remediate the latent safety threats (LST).

**Methods:**

This is a prospective cross-sectional study with a mixed method research design, which was conducted in the ED of Dhulikhel Hospital-Kathmandu University Hospital. The pretest questionnaire was used to determine baseline knowledge, attitude, and confidence of the staff. The ISS with minimal added cost was conducted involving multidisciplinary healthcare workers. The LSTs were recorded and appropriate remediation was performed. Voluntary post simulation feedback was collected after the sessions.

**Results:**

Overall 56 staff participated in at least one of the 35 simulation sessions, among which 45 (80%) responded to the questionnaires`. Twenty participants (45.5%) were reluctant to use the defibrillator. The self-reported confidence level of using defibrillator was low 29 (64.6%). The knowledge score ranged from 0 to 8 with the median score of 3 and a mean of 3.29 ± 1.8. There was no statistically significant difference in knowledge scores among participants of different occupational backgrounds, previous training, duration of work experience, and previous use of a defibrillator. A total of 366 LSTs {individual (43%), medication (17%), equipment (4%), and system/team (36%)} were identified (10.45 LST per ISS). The overall feedback from the participants was positive. Eighty percent of participants reported increased skills to use a defibrillator, and 82% reported increased confidence for managing such cases. They also agreed upon the need and continuity of such type of simulation in their workplace.

**Conclusions:**

The baseline knowledge score and the confidence level of the staff were low. Self-reported feedback suggested increased confidence level and teamwork skills after ISS. It promoted identification and remediation of latent safety threats. ISS serves as a cost-effective powerful educational model that can be implemented even in settings where finances and space are limited.

## Background

Medical education curriculum and methods of teaching have undergone significant changes all over the world. One of the reasons for the changes is concern for the patient’s safety [1, 2]. Simulation provides a means for trainees to acquire and practice clinical skills and competencies and to bridge the gap between theory and practice without direct contact with the patient. Issenberg et al. [1] define simulation as “a person, device, or set of conditions which attempts to present education and evaluation problems authentically. The student or trainee is required to respond to the problems as he or she would do under natural circumstances.” Simulation-based medical education (SBME) is a complex educational intervention and is well established as a practical, safe, structured, and effective training strategy to train health care professionals [2]. It is currently flourishing around the world as it provides trainees with the opportunity of facing real and/or rare clinical situations and intervenes with them across cognitive, procedural, communication, and teamwork skills [1–3]. Simulation allows learners to receive professional feedback through debriefing. With these opportunities, their technical and nontechnical skills are strengthened. The practice of simulation-based education is growing in Nepal. Some medical schools have institutionalized SBME into their curriculum and started to install and utilize simulation clinical skills laboratories for training. Despite the known evidence of effectiveness of SBME, its extensive application in resource-limited countries like ours is prohibited due to high cost. Installing an expensive simulation lab with high-fidelity simulators, duplicating the pattern in developed countries, may be futile where resources are constrained [4]. A dedicated simulation suite fully equipped with high-fidelity manikins and technology is very expensive, occupies permanent space, and cannot replicate the actual workplace environment. In a financially challenging situation, economic evaluation should be done before implementing simulation-based education [5].

In situ simulation (ISS) is defined as, “simulation that occurs in the actual clinical environment and whose participants are on-duty clinical providers during their actual workday” [3, 6]. The blending of simulation-based learning and real working environment helps address the problem of clinicians not being able to apply what they learn in actual work practice and thus may provide a powerful tool for continuing education [7]. Moreover, conducting a simulation in the actual working venue for learning purposes using available space, equipment, and resources will reduce the setup cost of the simulation lab and the need for dedicated space. Its feasibility and minimal cost may provide an opportunity to increase the number of institutions performing these realistic SBME even in rural and resource-limited settings. The fidelity (degree of realism) of a simulation is determined not only by high-fidelity expensive manikins, but also by psychological, environmental, and social fidelity which is very high during the ISS [8]. High-fidelity simulators are very expensive and not feasible in developing countries like ours; however, the ISS has the benefit of running a simulation at a low cost using an ordinary manikin and available resources, while increasing the fidelity of the simulation with psychological components as the learning context is more similar to the context of practice with participation of those personnel who normally work together as a team in real location and situations. Moreover, the indirect cost is low as the group of staff undergoing in situ training need not be released from the job. Another advantage of ISS is the identification of latent safety threats (LST). LSTs are system-based threats to patient safety that can occur at any time and are generally unrecognized by healthcare providers in day to day clinical practice which can be detected by ISS performed in real clinical settings [9, 10].

The health care professionals providing emergency patient care are required to manage complex cases and should be able to use defibrillators. In this study, we explored our experiences of ISS for unannounced tachyarrhythmia/cardiac arrest training in the ED using a team-based approach. We hypothesized that the implementation of ISS-based training with multidisciplinary approach would promote the trainee to acquire skills, practice teamwork with their own team members, and increase their confidence for managing tachyarrhythmia and using a defibrillator as well as improve the safety of the patients in emergency by identification of LSTs. In addition, this study aimed to highlight the cost effectiveness of this point of care simulation training which could be initiated without significant additional cost. This would serve as a model for widespread application of SBME globally where cost is the major hindrance.

## Methods

### Study design

This is a prospective cross-sectional observational study with a mixed method research design to explore the effectiveness of ISS involving multidisciplinary teams in ED with limited cost.

### Study setting and participants

The study was conducted in the ED of Dhulikhel Hospital-Kathmandu University Hospital with approximately 20,000 visits annually and has a high acuity level. The department is staffed by general practitioner (GP) faculty physicians, medical officers, intern doctors, nurses, and paramedics. In this ED system, patients are immediately triaged and unstable patients are directed towards the resuscitation room. The usual response team during this critical intervention includes faculty physician(s), medical officer(s), nursing staff(s), paramedic(s), and intern(s) all of whom are ED personnel. If needed, additional personnel from other departments are called upon for help. Thus, the normal care teams during resuscitation constitute 4–10 personnel depending on the time of the day. We implemented the ISS training targeting all interdisciplinary health personnel who respond to critical events in the ED’s resuscitation area. The faculty physicians (*n* = 5) were the facilitators for the simulation training and, therefore, were not included as the participants.

### Study variables and tools

1. *A pre-simulation questionnaire* was distributed among the different cadre of staff to explore their baseline knowledge and confidence in managing cases of tachyarrhythmia and the use of a defibrillator. A questionnaire was prepared by the authors encompassing 3 main domains:
  - Demography, professional qualification, and experience of the participants (7 items)
  - Awareness and attitude of the participants on defibrillator use (10 items)
  - Knowledge and practice of the participants related to tachyarrhythmia and use of a defibrillator (10 items) based on the 2015 American Heart Association (AHA) Advanced Cardiac Life Support (ACLS) Guidelines [11]. Knowledge assessment was done to assess the need for a subsequent theory class before the actual simulation.

The questionnaire was revised by 5 experts who were AHA certified instructors for ACLS for content and face validity (topic content and questionnaire construction). The validity of the questionnaire was further determined by piloting in a small similar mixed group (*N* = 10). After appropriate amendments, it was finalized for the study purpose.

2. *Form to record LST*. The LSTs were divided into individual, medication, equipment, and system/teamwork safety threats and recorded for each simulation by the faculty who conducted the simulation.
3. *Post simulation survey*. A voluntary post simulation survey form consisting of 10 items using a 5-tier Likert scale was collected to explore the perception of the participants towards the sessions.

### Study process and data sources

To determine the need of the training, a pretest was conducted which explored the baseline knowledge and confidence in managing cases of tachyarrhythmia and use of a defibrillator among the staff. A theory class (120 min) on the approach to tachyarrhythmia and cardiac arrest was delivered to all the participants. Before each simulation session, an assessment of clinical activities within the department was made to ensure minimal disturbance to ongoing patient care. The unannounced simulation was conducted in the resuscitation area involving multidisciplinary healthcare workers working during the shift or after the handover when the number of staff was relatively more. ED staff responded to the simulation event as they would normally do in a real patient scenario. The same participant could participate in more than one simulation. All personnel were required to participate in the simulation sessions**;** however, only those who completed the feedback form voluntarily were included in the study analysis. One feedback per participant was collected either at the end of posting (for interns) or towards the end of the study period voluntarily. All simulations were performed using an existing ordinary prototype manikin used for other training purposes (current price = 400 USD) and “SimMon” app (25 USD) in an iPad (belonged to one of the authors used for educational purpose, current price 700 USD) for generating the cardiac rhythms and vital signs. Standard ACLS scenarios of cardiac arrest and tachyarrhythmia were used for this purpose. The simulations were limited to 10–15 min with an equal or longer period of debriefing immediately after the sessions. A standardized ACLS debriefing template was used by the facilitators emphasizing self-reflection and reflection on the group performance. Video debriefing was not done due to cost and time factors. During the simulation, the LSTs were recorded in the predesigned form by the facilitator. During post simulation debriefing, individual and teamwork safety threats were communicated with the participant and the team. Applicable remediation was performed for the medicine and equipment LSTs.

### Statistical methods

Data was entered in Microsoft Excel and statistical analysis was performed using SPSS 23 version. Descriptive and frequency analysis was made for counts, percentage, and means or medians as appropriate to provide the overall picture of the responses. Mean score was compared for the duration of clinical experience, professional qualification, and previous training obtained by the participants by using ANOVA. *P* values < 0.05 were considered statistically significant. Latent safety threats identified during the in situ sessions were described and categorized qualitatively and were classified by the type of identified threat. Post simulation survey responses were collected, quantitative results were presented as descriptive frequencies and qualitative responses were categorized into themes and analyzed.

### Ethical clearance

Ethical approval was obtained from the institutional review committee. Written informed consent was obtained before a respondent completed the questionnaire. The questionnaire and surveys did not contain the name of the participants; thus, the confidentiality of the participants was maintained.

## Results

A total of 35 in situ simulation sessions were conducted over the period of 6 months. Seven scheduled simulation sessions had to be canceled either due to the presence of critical cases in the resuscitation area or overall high patient flow in ED. Overall, 56 staff participated in at least one of those simulation sessions with each participant participating in 2–4 ISS sessions in average. Forty-five participants (80%) responded to the questionnaires among which 17 (37.8%) were medical officers, 17 (37.8%) interns, 6 (13.3%) nurses, and 5(11.1%) paramedics. The age of 95.6% of the participants (*n* = 43) ranged from 20 to 29 years. Among all the participants, 23 (51.1%) were male and 22 (48.9%) were female.

Four participants (8.9%) did not know the location of the defibrillator in the ED, and 18 (40%) were not aware of the type of defibrillator (monophasic vs. biphasic). Twenty staff (45.5%) were reluctant to use the defibrillator in a patient if needed; the reason selected were “insufficient knowledge” and “fear of harm to patient” by 17 (85%) and 3 (15%) of them, respectively. The self-reported confidence level of using a defibrillator among the staff was explored. In the Likert scale of 5, 29 (64.6%) were not confident about the use of a defibrillator (Likert scale score of 1 and 2) while none of them were highly confident (Likert scale score of 5).

The knowledge score ranged from 0 to 8 (total score 10) with the median score of 3 and mean of 3.29 ± 1.8. The difference in knowledge scores among the different occupational background, previous training, duration of work experience, and previous use of a defibrillator is demonstrated in Table 1.

**Table 1:** The comparison of baseline knowledge score from the pretest questionnaire and different characteristics of the participants

Variables	*N* (%)	Mean score (SD)	*P* value (ANOVA)
Profession			
Medical officers	17 (37.8)	3.59 (2.238)	.539
Interns	17 (37.8)	3.12 (1.495)	
Nurses/paramedics	11 (24.4)	3.09 (1.640)	
Duration of work			
< 1 year	33 (73.3)	3.24 (1.786)	.469
1–5 years	11 (24.4)	3.27 (2.005)	
> 5 years	1 (2.2)	5	
BLS			
Yes	26 (57.8)	3.62 (1.899)	.082
No	19 (42.2)	2.84 (1.642)	
ACLS			
Yes	9 (20)	3.44 (1.236)	.541
No	36 (80)	3.25 (1.948)	
Defibrillator used previously			
Yes	7 (15.5)	3.71 (2.138)	.229
No	38 (84.5)	3.14 (1.735)	
Defibrillator use witnessed			
Yes	26 (57.8)	3.46 (1.86)	.804
No	19 (42.2)	2.93 (1.58)	

### Latent safety threats

**Fig. 1:**
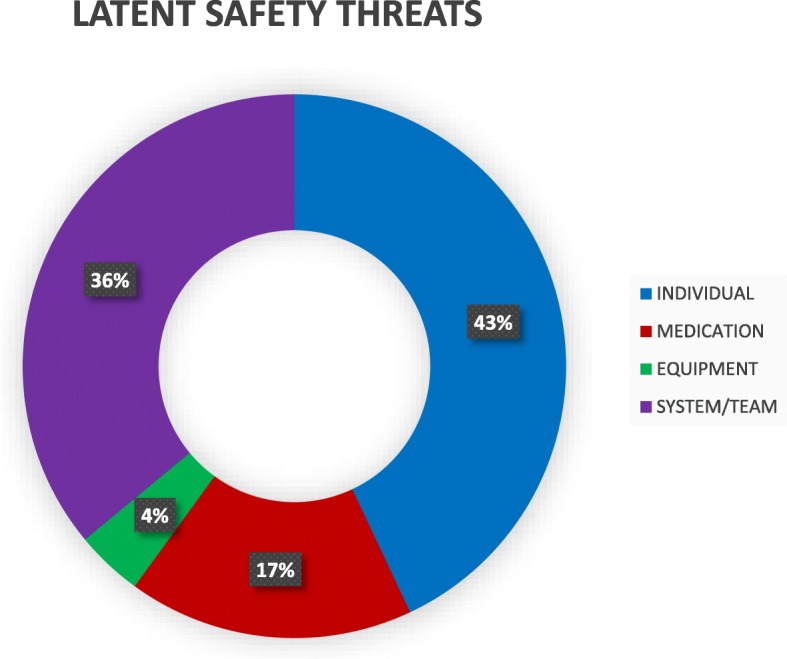
Latent safety threats

**Fig. 2:**
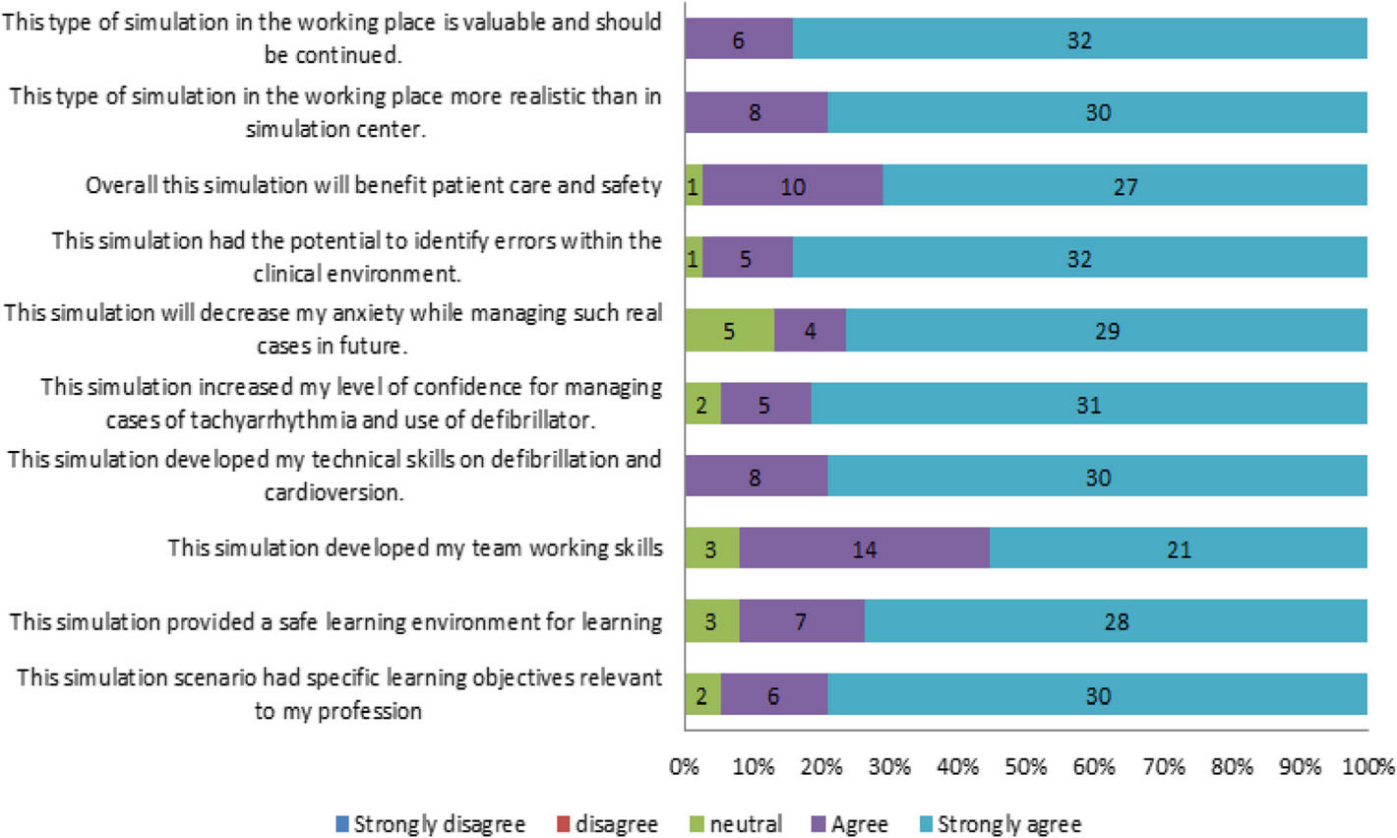
Feedback from the participants

A total of 366 LSTs were identified and are described in Fig. 1. This resulted in an identification rate of 10.45 latent threats for every ISS performed (Table 2). The feedback form was returned by 38 (85%) participants and is illustrated in Fig. 2.

**Table 2:** List of latent safety threats

Type	Description	*N*
Individual	Delay in checking response	4
	Delay in assessing central pulse	6
	Incorrect carotid pulse assessment	2
	Prolonged carotid pulse assessment	1
	Delay in calling for help	2
	Delay in initiation of chest compression	10
	Poor compression quality	12
	Interruptions during CPR	27
	Inadequate ventilation	6
	Too frequent pulse checks	13
	Pulse checks while continuing compressions	11
	Delay in putting the monitor and assessing rhythm	9
	Unable to recognize rhythm	2
	Delay in defibrillation	16
	Inappropriate positioning of pads	8
	Difficulty familiarizing with defibrillator	7
	Inappropriate energy selection	6
	No change in compressor roles	9
	Unsafe delivery of shock	6
	Total	157
Medications	Delay in opening IV line	3
	Inappropriate dose of adrenaline	6
	Error in recognizing adrenaline ampoule	1
	No flush after adrenaline	10
	Too late administration of adrenaline	8
	Too frequent administration of adrenaline	6
	Too seldom administration of adrenaline	5
	Too late administration of amiodarone	17
	Wrong dosing of amiodarone	8
	Total	64
Equipment	Suction not working	2
	Delay in initiation of defibrillator	3
	Amiodarone unavailable	1
	No hard board for chest compression	2
	Lubricant not available immediately	5
	Total	13
SystemTeam	Clear roles and responsibilities not assigned	15
	Lack of closed loop communication	19
	Uneven Job allocation	11
	Team leader should hands off from any work when possible	9
	Not summarizing	22
	No orientation to new team member	12
	No transparent thinking	24
	Open indirect communication	20
	Total	132
	Grand total	366

## Discussion

### Cost

In contrast to traditional simulation, which is conducted at a simulation center out of hospital or in hospital at a different setting, we conducted simulation in the actual working venue in our ED resuscitation area using available equipment and resources with minimal additional cost. A simulation does not have to be in a separate expensive simulation suite or a simulator does not have to be an expensive electronic simulator to attain high fidelity. Our ISS executed well with prototype simulator and available resources and provided realistic simulation experience at a low cost (total cost = 1125 USD); in addition, the psychological and environmental fidelity was high. With meticulous planning, these ISS were able to provide team training for the entire department and test the patient safety by detecting LSTs at minimal expense. The dedicated simulation suites are expensive to equip, occupy space, and cannot completely replicate the actual working environment despite the high-fidelity simulator. Taking simulation to the working environment reduced both fixed cost (space, manikins, furniture, defibrillator, and other equipment) and variable costs (medications, payment for the trainees, and other consumables) that are very high in a dedicated simulation suite. Moreover, the participants were not needed to be called away from their clinical work as the simulation sessions were conducted during their duty hours inside their working station. This also has an indirect implication on the cost as the participants can be trained without causing the operational disruption caused by the release of the team from the work for the training purpose. The budget for future ISS can further be minimized (400 USD for manikin) by excluding the use of an iPad and associated application like SimMon as the vital signs can be verbalized by the facilitator and the cardiac rhythms can be demonstrated in a laptop or printed on a paper. The commitment of the faculties to train their staff and the team dynamics among the staff demonstrate that high-fidelity simulation training can be possible despite financial limitations. The markedly reduced cost of ISS in comparison to the simulation at the dedicated simulation site, with preservation of the training objective and fidelity, provides a model to overcome the financial barriers that prevent the practice of SBME by many institutions nationally and internationally.

### Knowledge

This study showed that the baseline knowledge score about the use of a defibrillator was overall low (mean 3.29 ± 1.8) and the difference was not significant among the different occupational backgrounds, previous training, duration of work experience, and previous use of a defibrillator. A theory class was organized for those participants with a low score before the simulation sessions for better understanding of the cardiac rhythms and its treatment. Almost half of the health personnel were reluctant to use a defibrillator. The self-reported confidence level of the staff of using defibrillator was very low. These critical findings reinforce the need of such training initiatives to the novice doctors and paramedical staff in our settings. The majority of participants (80% strongly agreed and 20% agreed) reported increased technical skills in their ability to use a defibrillator. Five percent of participants were neutral, 13% agreed, and 82% strongly agreed that the simulation increased confidence for managing cases of tachyarrhythmia and use of a defibrillator. This project promoted simulation-based training in the ED with multidisciplinary involvement as a method to improve clinical care and confidence level. A previous prospective in situ simulation intervention to improve arrhythmia detection within the ED demonstrated that arrhythmia detection rates increased from 5 to 55% [12]. A systematic review by Rosen et al. [7] found 29 studies of in situ simulation and suggested positive impacts on learning and organizational performance. An observational study [13] found that survival rates for cardiopulmonary arrest increased to approximately 50% (*p* = .000), in correlation with an increased number of mock codes (*r* = .87). Steinemann et al. (2011) [14] did a before-and-after study of in situ-based trauma team training, found that teamwork ratings, task speed, and task completion rates improved within the simulations, and showed that these benefits translated into clinical practice.

### Team work

Communication is frequently found to be at the root of human errors and adverse events [15] making it an important topic as staff learn through the simulation of these emergency events. In our setting, in situ simulation also provided a means to continuously identify and reinforce communication and teamwork skills (132 communication problems identified). A total of 92% of the responders (55% strongly agreed and 37% agreed) claimed that the simulation session developed the team work skills. Miller et al. (2012) [16] used a pre-/post-observational design using multidisciplinary in situ simulation translated to improvements during real trauma resuscitations across 12 of 14 nontechnical skill components, though only communication showed statistical significance.

### Latent Safety Threats

The use of in situ simulation accomplished the dual goals of providing continuous opportunities to deliberately practice technical and nontechnical skills as well as identifying and remedying LSTs. Ninety-seven percent of the respondents agreed that this simulation had the potential to identify errors within the clinical environment. Patterson et al. (2013) studied 90 ISS events in an urban ED over a 12-month period and found that a latent safety threat was identified at a rate of one in every 1.2 simulations performed [8]. During this study period, a total of 366 LSTs were identified (identification rate of 10.45 latent threats for every in situ simulations performed) which was very high in comparison to the previous studies. Active failures by clinicians due to knowledge deficits (43%) were the highest among them. Teamwork deficit was also commonly noted (36%). The probable reasons behind the high knowledge deficit would be that 37.8% were interns who are freshly graduated physicians who have limited clinical exposure, 73.3% had < 1 year of clinical experience, and only 15.5% had previously used a defibrillator. The senior faculty physicians were excluded from the study as they were the facilitators for the sessions. The teamwork LST was also high as teamwork communication is not regularly practiced earlier due to lack of simulation training prior to this study. There was rapid turnover of the interns (every 1 month) and medical officers (6 months–1 year); therefore, we could not demonstrate the statistical significance regarding the decrease in the LSTs in relation to the number of ISS. LST detected during training were documented and addressed, and efforts were made to address the individual and teamwork LSTs immediately after the ISS during debriefing. The medication and equipment LSTs noted were reported to the concerned personnel in the ED and mitigated if feasible. For example, the lubricant jelly was made available near the defibrillator; the suction machine was repaired after simulation. These actions not only led to the recognition of the knowledge, equipment, and communication gaps but also provided a means to reinforce the measures to close the gaps.

The theme of fidelity and safety reflected in the feedback provided by the participants. All participants were positive about the statement that this type of simulation in the working place is more realistic than in the simulation center, and 92% agreed that this provides a safe learning environment. All of them also stated that this type of simulation in the working place is valuable and should be continued in the future.

### Limitations

Post intervention knowledge was not studied and compared with the pre-intervention score as the participants were not randomized into different categories of training (in situ vs. off-site or theory vs. simulation). The theory class was given to the participants as the baseline knowledge was found to be low. Participants were not divided into theory vs. simulation group as this was not our objective. Our objective was to provide all staff with experience of ISS to enhance their technical and nontechnical soft skills. Posttest outcome would reflect not only the effect of ISS, but also the theory class. So we realized that if a post test was conducted, the result would have been confounded by the effect of theory class on final posttest scores. In reference to the Kirkpatrick’s four levels of evaluating training programs [17], this study evaluated only the first step—“evaluating reactions” from the participants. A skill-based score could have been a better option for the evaluation of the second level—“evaluating learning” of the participants. The third level—“evaluating behavior”—was evidenced only by self-reporting. However, this could have been documented in a validated teamwork scale. The fourth level—“evaluating results”—needs time and more effort. We are expanding our experience with ISS and plan to do further evaluation on results in the future.

Some ED staff also raised concerns regarding the impact of in situ simulations on patient care, and there were instances where the planned simulation sessions needed to be canceled due to the high flow of patients or arrival of a sick patient in the resuscitation area. Performance anxiety of healthcare providers posed a significant challenge initially as they were being observed by their peers and their seniors.

## Conclusions

The baseline knowledge score and the confidence level of the staff were low. The implementation of in situ simulation-based training in an actual clinical environment with multidisciplinary approach demonstrated the self-reported perceived improvement in confidence and teamwork skills to respond to an emergency situation. It promoted identification of latent safety threats and therefore has the potential to significantly improve the safety of patients. Considering the low cost of running the simulations with the available resources and the positive feedback from the participants as well as improved safety of the patients in emergency by identification of LSTs, ISS should be implemented extensively as a useful educational technique in every hospital and in every department even when finances are stretched and space for simulation suites are not available.

Future efforts should focus on the implementation of ongoing ISS as quality improvement projects and inclusion of the ISS in the curriculum incorporating other critical care scenarios and environments. Further research should be performed to demonstrate the transfer of the skills gained by the ISS into the real-life setting encompassing all four levels of evaluating a training program. The next step of this project would be to explore whether the experiential learning afforded by this process provided an opportunity to transfer these skills into the real-life setting by documenting the improvement in knowledge and skills.

## Data Availability

The dataset generated and analyzed during the current study is available from the corresponding author on request.

## Abbreviations

ACLS: Advanced cardiac life support
AHA: American Heart Association
ED: Emergency Department
GP: General Practice
ISS: In situ simulation
LST: Latent safety threats
SBME: Simulation-based medical education
